# A New Approach to Implementing 3D-Printed Material Structures for Protective Gloves with the Use of Ultrasonic and Contact Welding Processes: A Preliminary Study

**DOI:** 10.3390/ma17225404

**Published:** 2024-11-05

**Authors:** Emilia Irzmańska, Agnieszka Cichocka, Adam K. Puszkarz, Olga Olejnik, Paulina Kropidłowska

**Affiliations:** 1 Department of Personal Protective Equipment, Central Institute for Labour Protection–National Research Institute, 16 Czerniakowska Str., 00-701 Warsaw, Poland; emirz@ciop.lodz.pl (E.I.); pakro@ciop.lodz.pl (P.K.); 2 Institute of Textiles Architecture, Faculty of Material Technologies and Textile Design, Lodz University of Technology, 116 Żeromskiego Str., 90-543 Lodz, Poland; agnieszka.cichocka@p.lodz.pl; 3 Textile Institute, Faculty of Material Technologies and Textile Design, Lodz University of Technology, 116 Żeromskiego Str., 90-543 Lodz, Poland; adam.puszkarz@p.lodz.pl

**Keywords:** contact welding, ultrasonic welding, thermoplastic polymers, protective materials, PPE, protective gloves

## Abstract

This study presents a new approach to developing protective material structures for personal protective equipment (PPE), and in particular for protective gloves, with the use of ultrasonic and contact welding processes. The goal was to assess the quality of joints (welds) obtained between a synthetic polyamide knitted fabric (PA) and selected polymers (PLA, ABS, PET-G) in the developed materials using X-Ray microtomography (micro-CT). Quantitative and qualitative analyses were performed to determine the joint area produced by the selected welding methods for the examined materials. In this article, we assumed that obtaining a greater contact area seems to be the most promising from the point of view of future PPE utility tests characterizing protective glove structures. This research is a continuation of our previous study focused on functional 3D-printed polymeric materials for protective gloves.

## 1. Introduction

The structure of protective materials plays a key role in the performance of personal protective equipment (PPE). Such materials must meet a variety of requirements, depending on the environment in which they are used and the type of hazards they are designed to protect against. Various aspects of the structure of protective materials affect their functionality and performance [1,2,3]. The composition of materials such as synthetic polymers (Kevlar, Nomex, or ultra-high molecular weight polyethylene—UHMWPE) offers high tensile strength, abrasion resistance, and flame retardancy [4]. In the case of protective gloves, of great importance are coatings and laminates attached to their external surface and containing substances that increase their resistance to chemicals, water, oils, and other hazardous substances [5]. As regards the internal structure, materials such as woven or knitted fabrics are often used in clothing that protects against cuts and impacts, as they can offer greater flexibility and freedom of movement in dynamic work environments where their mechanical properties are crucial. Materials with high tensile and tear strength are essential in PPE to provide long-term protection against physical damage. Puncture resistance is critical in protective gloves and clothing used in tasks involving sharp tools or equipment [6,7,8]. Thermal properties, flame resistance, and thermal insulation are important for firefighters exposed to extremely hot temperatures. Chemical resistance is of the essence for materials exposed to a wide range of substances, including acids and solvents, to protect the wearer from contact with them [9]. Finally, in terms of biological safety, protective materials provide a barrier to pathogens, which is important for medical personnel and laboratory workers [10,11].

However, it is not only the protective properties of PPE but also its ergonomic qualities that are important for the users. For instance, the construction and thickness of glove materials, which are usually related to the number of their constituent layers, determine the manual dexterity offered by protective gloves [12]. Various additional elements, such as seams, may also hamper work performance. Therefore, novel processing methods should be developed to avoid such impediments. It must also be noted that the fit of PPE has a statistically significant effect on work performance. Well-designed and well-fitted protective clothing, gloves, footwear, etc. can contribute to fast reaction times, a high range of motion and mobility, and satisfactory muscle activation and lung function as well as acceptable endurance and tolerance [13]. Therefore, it is not only the design of materials and equipment but also preparation methods that have a significant impact on PPE performance parameters.

Ultrasonic welding may be proposed as one of the joining methods that can be used in materials potentially suitable for PPE. In this method, high frequency acoustic vibrations (20–40 kHz), which are beyond the range of human hearing, are applied to induce low amplitude mechanical vibrations (20–30 µm). In this way, the interfaces of PPE materials can be quickly joined exclusively due to welding pressure without the need for a traditional sewing process. Paper [14] investigated the effects of fabric structure and ultrasonic welding on the performance of spunlace surgical gowns to compare the behavior of fabrics with and without stitches in terms of draping, air permeability, and surface friction. The study proved that sewing reduces air permeability by increasing fabric thickness in the area of welds (seams). Jevšnik et al. [15] examined the effects of ultrasonic welding parameters on bond strength, seam thickness, and stiffness as well as water permeability in two types of four-layered fabrics suitable for the inner parts of sport shoes. The study results as well as data from the shoe manufacturer show that all the produced welded seams provided the necessary bond strength. This aspect is also important and should be considered in the case of other PPE materials, including gloves composites.

The contact welding process, or more specifically hot-plate welding (hot pressing), is also attractive from the point of view of preparing PPE materials. This method does not require traditional sewing either. It involves pressing the elements to be joined against a heated, usually flat plate. The elements are removed when they have melted sufficiently, and the resulting joined material is cooled down [16,17]. Similar to ultrasonic welding, contact welding results in thermal bonding, where heat and pressure are utilized to soften and fuse materials. The molten materials act as adhesives and become stable below the melting temperature [18]. This method can be used to produce not only multi-layered composites to be incorporated in innovative materials for safety equipment but also fiber-reinforced composites for ballistic resistance purposes [18,19,20]. Yan Hong et al. [21] found that hot pressing is even more advantageous than electrospinning in preparing filtration textiles for protective masks.

Both welding methods (hot-plate and ultrasonic) have the potential to replace traditional sewing in protective materials thanks to their numerous benefits. In some cases, the described methods are more suitable for improving mechanical properties of PPE, where traditional sewing is insufficient. For instance, extra reinforcement in the form of additional thermoplastic items incorporated into different parts of gloves is needed to obtain satisfactory protective properties, such as a high level of cut resistance [7]. However, it is important to consider the type of polymer used, as not all thermoplastics are compatible with ultrasonic welding. The size of the welded elements is also important [17]. PPE made of materials prepared via the presented welding methods without stitches can ensure safety and comfort to workers in different environments. This study is a continuation of our previous research describing functional 3D-printed composites for protective gloves [7]. This paper presents a preliminary study of welding methods that can be used for creating complex material structures with potential applications in protective gloves. Herein, we are focused on X-Ray microtomography (micro-CT) as the main tool for assessing the quality of the obtained welds, which may help in selecting the proper material and suitable welding method to create cut-resistant material dedicated to protective gloves. Based on the results, we are going to choose one polymer to confirm how mechanical properties are related with the obtained observations of welds.

## 2. Materials and Methods

### 2.1. Materials and Sample Preparation Methods

In optimizing ultrasonic and contact welding processes for protective glove materials, many factors need to be taken into account, such as material type and thickness, vibration frequency and amplitude, welding time, and pressure. Tests must be conducted for different material samples to identify the optimal parameters for durable and aesthetic joints that meet user safety and comfort requirements.

**Figure 1:**
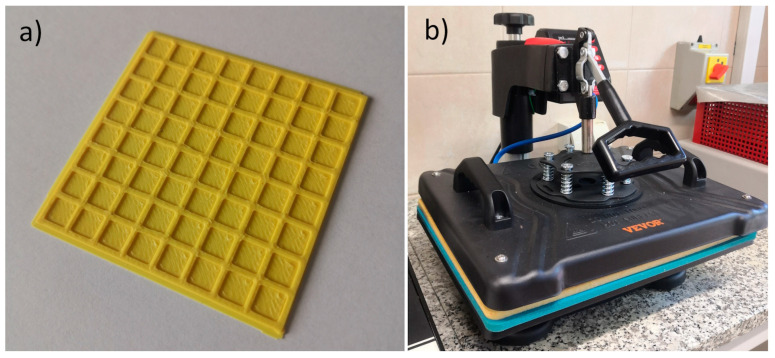
(**a**) Example of a thermoplastic sheet (ABS) with a square grid pattern on one side. (**b**) Heat transfer machine used for contact welding.

The present study used three polymeric and copolymeric sheets made of polylactide (PLA), poly(ethylene terephthalate)-glycol (PET-G), and acrylonitrile-butadiene-styrene (ABS). These 0.5 mm thick sheets were 3D printed using fused deposition modeling (FDM) courtesy of the company SMK3D Jakub Saramak (Chechło Pierwsze, Poland). The applied 3D processing parameters are given in Table 1. The filaments used in this study were commercially purchased from Fiberlab S.A. (Brzezie, Poland), and included PLA (trade name: Fiberlogy EASY PLA, *ρ* = 1.24 g/cm^3^), PET-G (trade name: Fiberlogy EASY PET-G, *ρ* = 1.27 g/cm^3^), and ABS (trade name: Fiberlogy EASY ABS, *ρ* = 1.04 g/cm^3^). All of these filaments are thermoplastic, which means that they soften above a certain temperature and harden after cooling without compromising the quality of the material. The prepared sheets were characterized by a square grid pattern on one side (Figure 1a) to improve welding efficiency.

The synthetic polyamide (PA) knitted fabric (S. I. Zgoda, Konstantynów Łódzki, Poland) used in this study had a basis weight of 50 g/m^2^.

Thermoplastic sheets were joined to the fabric using two methods, i.e., contact and ultrasonic welding, which were then compared using X-Ray microtomography (micro-CT) to assess the quality of the resulting welds.

Both ultrasonic and contact welding were found to result in seamless waterproof joints ideal for protection against liquids and microorganisms. The speed and efficiency of the production process are additional advantages, with possible applications in the medical, food, and pharmaceutical sectors. Taking into account the design flexibility of the method, it can be used to develop advanced and comfortable protective gloves.

#### 2.1.1. Contact Welding Method

Contact welding (the hot-plate method) was performed using a multi-function digital heat transfer machine that joined 3D-printed thermoplastic plates with PA knitted fabric. This study used a TLM13135 press from VEVOR (Yiwu City, China) equipped with a heating platen covered with poly(tetrafluoro ethylene) (PTFE) (Figure 1b). The working temperature of the platen was 200 °C. First, the knitted fabric was placed on a stationary table at room temperature. Next, the thermoplastic plate was placed on the fabric. The plate and the fabric were covered with baking paper to prevent the press from contamination by molten polymer. Finally, the hot platen was placed on the material and pressed by the clamp for 35 s. The same parameters were applied for all samples (PLA, PET-G and ABS thermoplastic plates). The finished materials were investigated by micro-CT.

#### 2.1.2. Ultrasonic Welding Method

**Figure 2:**
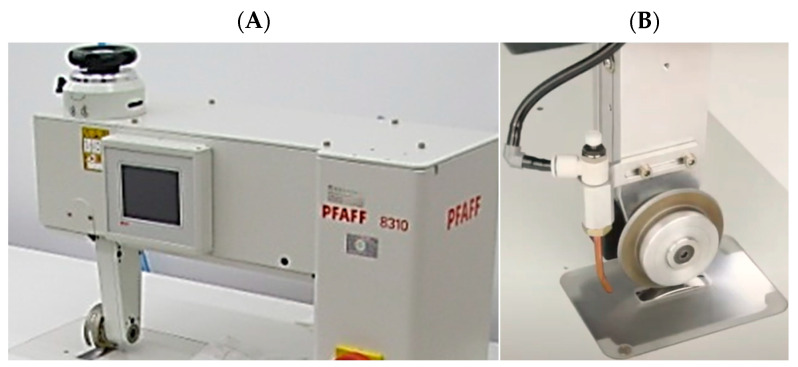
Programmable ultrasonic welding machine (**A**) with a bottom sonotrode (**B**) from PFAFF 8310.

The ultrasonic welding machine used in the tests was a PFAFF 8310 device (PFAFF, Kaiserslautern, Germany). (Figure 2A), in which the workpiece is held between the sonotrode and the anvil wheel (Figure 2B) and welded continuously under pressure. In the course of ultrasonic welding, the material is subjected to rapidly changing pressure vibrations that generate heat beneath its surface. Ultrasonic welding is a modern, innovative, and economical alternative to traditional sewing with thread. It is especially suitable for joining laminated, coated, and high-polymer-content clothing fabrics as well as technical nonwovens.

The selected operating parameters were as follows: advance speed of 1.7–2.5 m/min and pressure of 0.2–1 bar for a 5 mm weld width, as determined by preliminary experimental investigations. A flat anvil wheel was chosen for welding the test samples, considering the application area of the protective materials.

### 2.2. Research Methods

High-resolution X-Ray computed tomography was conducted using a SkyScan 1272 device (Bruker, Kontich, Belgium) to calculate the contact area of polymeric sheets (PET-G, PLA and ABS) with the polyamide knitted fabric. The scanning conditions were as follows: X-Ray source voltage of 50 kV, X-Ray source current of 200 µA, pixel size of 5.5 µm, and 180° rotation with a step of 0.2° without a filter. Two-dimensional scans were processed into 3D reconstructions of the examined materials using NRecon v.1.6.0 software (Bruker, Kontich, Belgium), and the knitted fabric-welding contact area was transformed in CTAn v1.14.4 software (Bruker, Kontich, Belgium). Three-dimensional visualization was performed by means of CTvol v.2.3.2.0 software (Bruker, Kontich, Belgium).

## 3. Results

### X-Ray Microtomography (Micro-CT)

Quantitative and qualitative analyses of the objects examined using the micro-CT is possible due to the contrast between their constituent structures resulting from differences in density and composition causing corresponding differences in X-Ray absorption. The X-Ray absorption of the knitted fabric is lower than that of the polymer sheets, which enables the identification and evaluation of the elements of the scanned objects (the surrounding air absorbs almost no X-Rays). Micro-CT has been successfully used by the authors to study the geometry of textiles for clothing [22] and technical [23] and pharmaceutical applications [24].

**Figure 3:**
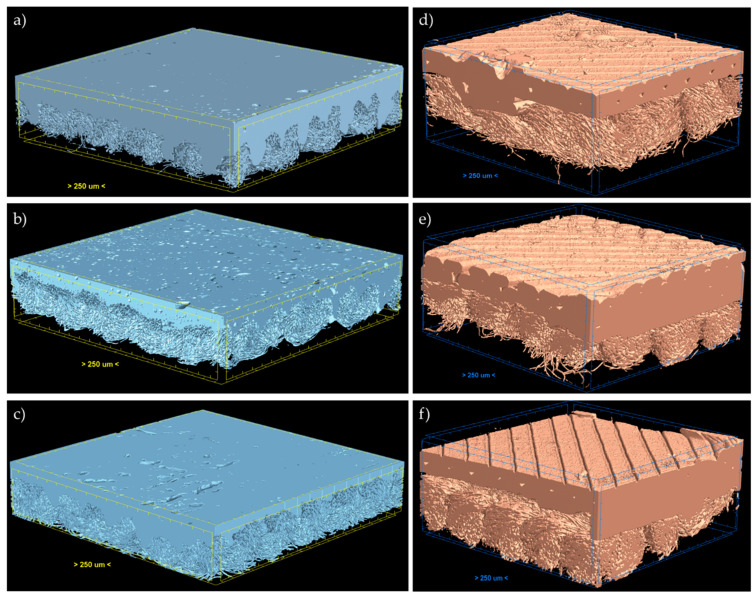
3D micro-CT visualizations of joints between three polymer samples (PLA, PET-G, ABS) and the knitted fabric for both joining methods (contact welding: (**a**–**c**); ultrasonic welding: (**d**–**f**)).

As can be seen from Figure 3, the entire surface of the knitted fabric adhered to the produced polymer welds in both joining methods (contact and ultrasonic welding).

According to the proposed approach to developing protective material structures, the present study did not determine adhesion force, which refers to the force of attraction between the surfaces of different materials. Adhesion force is a measure of how well one surface adheres to another, which in the context of protective materials is difficult to ascertain due to measurement uncertainty. Adhesion results from various molecular forces, including van der Waals forces, hydrogen bonds, and electrostatic interactions, which can be studied using atomic force microscopy (AFM) or force spectroscopy. A high adhesion force means that surfaces are strongly attracted to each other and are difficult to separate. A low value indicates that the surfaces separate easily. Although the measurement of adhesion force is crucial in the design of materials and technological processes where joint reliability and durability is critical, it does not provide sufficient certainty from the point of view of the mechanical loads that PPE materials may be subjected to during occupational tasks. From this point of view, the analysis of contact area between joined materials (especially when they are as disparate as those considered herein) seems interesting.

In the present study, contact welding resulted in the partial melting of all three polymers used, causing them to penetrate into the structure of the knitted fabric. The largest amount of polymer penetrated through loops of the polyamide knitted fabric in between yarns. However, some of the polymer penetrated into the knitted fabric also through the yarn in places where the fiber structure was the loosest (weakened yarn). At the adopted parameters, ultrasonic welding (by contrast to contact welding) did not cause considerable melting of any of the three polymers.. The partial polymer melting obtained by contact welding resulted in an increased contact area between the knitted fabric and welds (better adhesion) as compared to that of the ultrasonic method, where contact area results solely from the topography of welds in contact with the knitted fabric. Table 2 shows the percentage expansion of the contact area for both tested methods. For ultrasonic welding, the flat surface area of polymer welds before partial melting served as a reference (100%). In the case of contact welding, the reference was the flat (undeformed) polymer sheet surface prior to the experiment (100%).

**Figure 4:**
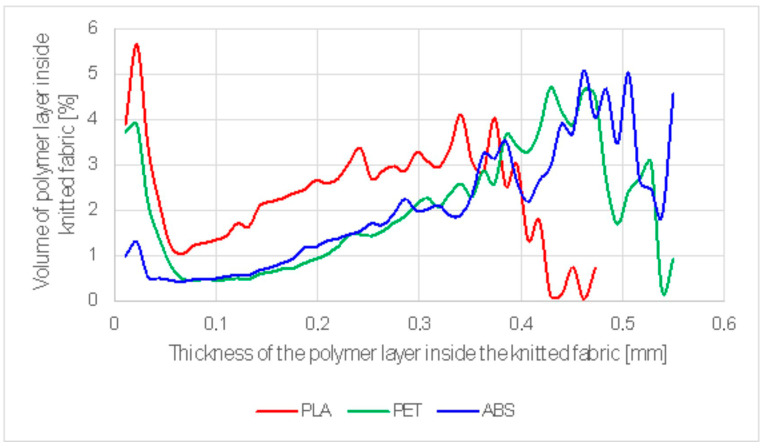
Depth distribution of polymer penetrating into the knitted fabric in contact welding.

The depth distribution of the polymers that penetrated into the knitted fabric as a result of contact welding is shown in Figure 4. Table 3 shows the mean penetration depth, which was 0.22768 mm for PLA, 0.33283 for PET-G, and 0.37091 for ABS.

**Figure 5:**
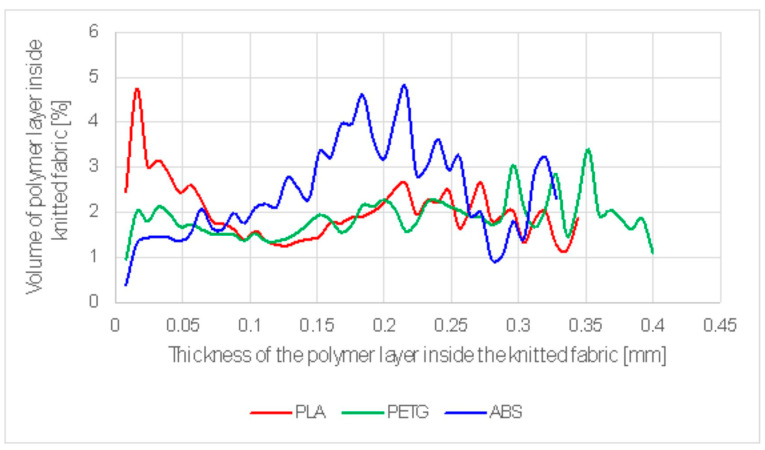
Thickness distribution of polymers that penetrated into the knitted fabric as a result of ultrasonic welding.

The depth distribution of the polymers that penetrated into the knitted fabric due to ultrasonic welding is given in Figure 5. The mean penetration depth was 0.19104 mm for PLA, 0.22928 mm for PET-G, and 0.18275 mm for ABS, as shown in Table 4.

**Figure 6:**
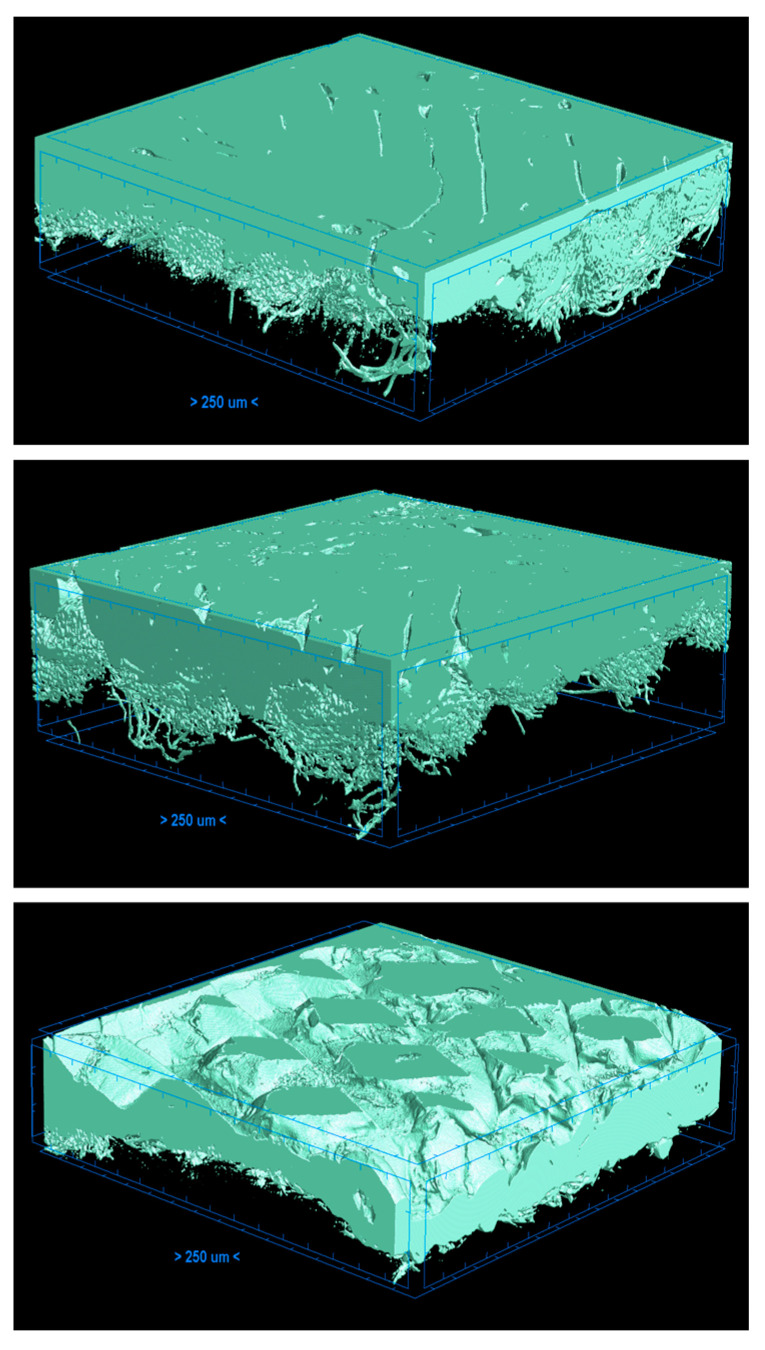
3D micro-CT visualizations of joints of three polymer welds (PLA, PET-G, and ABS from top to bottom, respectively) for ultrasonic welding.

The results of ultrasonic welding with modified parameters (advance speed decreased by 30%), which caused partial polymer melting, are presented in Figure 6.

The analysis of ultrasonic welding with modified parameters (UWMP), involving the partial melting of polymers and their better integration with textile structure, reveals a reduced thickness of the polymer layer (Table 5). At UWMP, the contact area between the knitted fabric and the PLA was significantly higher as compared to that of the first tests of ultrasonic welding (an increase in percentage expansion from 373% to 1238%). For PET-G, the increase in contact area expansion was from 660% to 856%, while for ABS, the contact area slightly decreased from 436% to 400%.

## 4. Discussion

Adhesion is a key phenomenon that allows materials to be joined in various technological and industrial processes. Adhesion between two materials involves the attraction and bonding of their surfaces. It is a process in which intermolecular forces cause those materials to stick together at the molecular level. Adhesion plays a key role in many fields, such as engineering [25], chemistry [26], biology [27], and medicine [16,28]. Factors that affect adhesion include intermolecular forces, such as van der Waals forces, hydrogen bonds, and electrostatic interactions. In addition, high surface energy usually indicates the ability to create better adhesion because it enables the creation of stronger intermolecular bonds (which is outside the scope of this study). However, when designing the structure of protective clothing materials, a compromise must be struck between strong intermolecular bonds and the integration of protective coatings and welds with the textile carriers—the main elements of protective clothing, which need to remain flexible and capable of bending and draping over the user’s body. In this context, research was continued on ultrasonic welding to increase the plasticization of the tested polymer welds and the resulting contact area.

Qualitative and quantitative assessments of the obtained welds were conducted by means of high-resolution X-Ray computed tomography, which is an attractive non-destructive method of evaluating thermoplastic/fabric joints. This aspect is relevant for PPE materials, including protective glove composites, where reliability, safety, and ergonomic properties are the most important [12,13]. Such assessments may also help determine the compatibility of a given thermoplastic with a given welding method. Micro-CT has been recently applied in a variety of areas, including the geometry of clothing textiles [22] as well as technical [23] and pharmaceutical applications [24]. This examination method holds a large potential for assessing contact areas between different PPE materials, and in particular within protective glove composites. For instance, information obtained from micro-CT may help improve the prepared composites by optimizing welding methods to enhance welds at the thermoplastic/fabric interface.

The idea of increasing contact area was considered in connection with the porous structure of the knitted fabric, so that the combination of polymeric material with the fabric would result in a greater penetration of the protective layer (polymer) into the fabric structure. The resulting composites could be used in PPE, including protective gloves. This study applies ultrasonic welding given its advantages, such as seamless and waterproof joints, which are ideal for protection against liquids and microorganisms. The anvil wheel of the welding machine, often also called the pressure wheel, is an important element in the welding process and especially in resistance welding (roller welding). Anvil wheels play a key role in ensuring appropriate contact, pressure, and flow of electric current through the welded materials. Our considerations assume that the shape of the anvil wheel and its grooves will allow for greater flexibility of ultrasonic welds, taking into account the properties of the polymers used (which solidify upon cooling). At the same time, the contact area between the polymers and the knitted fabric is responsible for the durability of the joint; the larger it is, the stronger the joint. It seems reasonable to continue further research focusing on functional testing of protective clothing depending on its intended purpose—e.g., protection against cuts.

## 5. Conclusions

This research continues our previous investigations into functional 3D-printed composites for protective gloves [7]. The current paper is focused on two different welding processes that can be used in the production of PPE materials, including protective glove materials. Both contact and ultrasonic welding have their unique advantages and potential in this respect. The choice of method depends on the specific strength, impermeability, and flexibility requirements as well as the type of hazards against which the clothing is protecting. Well-designed protective materials made using the studied welding methods can significantly increase user safety and comfort in a variety of work environments. However, the choice of the welding method depends on the applied materials because not every thermoplastic is compatible with both of them, which affects adhesion performance. The present research revealed that the contact area between the PA knitted fabric and the 3D-printed protective polymeric sheet made of PLA was higher for ultrasonic welding. In the case of PET-G and ABS, the contact area values obtained by ultrasonic welding were lower than those for contact welding. Research will be continued to investigate the durability and quality of the obtained joints between polymeric layers and knitwear, taking into account the developed structure of the protective material intended for applications in protective clothing. Further studies are also planned to examine the prepared composites in terms of protection against mechanical damage such as cuts.

## Figures and Tables

**Table 1:** 3D printing parameters for the selected polymer and copolymer materials.

Material	Print Temperature [°C]	Bed Plate Temperature [°C]	Print Speed [mm/s]
PLA	215	60	70
PET-G	245	85	60
ABS	245	90	60

**Table 2:** Percentage expansion of contact area between the knitted fabric and polymer welds for ultrasonic and contact welding methods.

Polymer Type	Ultrasonic Welding (in %)	Contact Welding (in %)
PLA	373	282
PET-G	660	1264
ABS	436	528

**Table 3:** Mean and SD values of polymer penetration into the knitted fabric as a result of contact welding.

Polymer Type	Mean [mm]	Standard Deviation [mm]
PLA	0.22768	0.12514
PET-G	0.33283	0.15288
ABS	0.37091	0.13458

**Table 4:** Mean and SD values of polymer penetration into the knitted fabric as a result of ultrasonic welding.

Polymer Type	Mean [mm]	Standard Deviation [mm]
PLA	0.19104	0.11942
PET-G	0.22928	0.12362
ABS	0.18275	0.08143

**Table 5:** Changes in the percentage expansion of contact area between the knitted fabric and welds for the original and modified ultrasonic welding methods.

Polymer Type	Ultrasonic Welding Method (in %) ^1^	UWMP Ultrasonic Welding Method (in %) ^1^
PLA	373	1238
PET-G	660	856
ABS	436	400

^1^ The flat surface area of polymer welds before partial melting served as a reference (100%).

## Data Availability

The original contributions presented in the study are included in the article material, further inquiries can be directed to the corresponding author.
